# A Taxonomy for Augmented and Mixed Reality Applications to Support Physical Exercises in Medical Rehabilitation—A Literature Review

**DOI:** 10.3390/healthcare10040646

**Published:** 2022-03-30

**Authors:** Benjamin Butz, Alexander Jussen, Asma Rafi, Gregor Lux, Jens Gerken

**Affiliations:** 1 Institute for Innovation Research and Management, Westphalian University of Applied Sciences, 44801 Bochum, Germany; 2 Human-Computer Interaction Group, Westphalian University of Applied Sciences, 45897 Gelsenkirchen, Germany; alexander.jussen@w-hs.de (A.J.); jens.gerken@w-hs.de (J.G.); 3 Computer Graphics Group, Westphalian University of Applied Sciences, 45897 Gelsenkirchen, Germany; asma.rafi@w-hs.de (A.R.); gregor.lux@w-hs.de (G.L.)

**Keywords:** mixed reality, augmented reality, extended reality, virtual reality, visualization, visual cues, medical, rehabilitation, therapy, exercise

## Abstract

In the past 20 years, a vast amount of research has shown that Augmented and Mixed Reality applications can support physical exercises in medical rehabilitation. In this paper, we contribute a taxonomy, providing an overview of the current state of research in this area. It is based on a comprehensive literature review conducted on the five databases Web of Science, ScienceDirect, PubMed, IEEE Xplore, and ACM up to July 2021. Out of 776 identified references, a final selection was made of 91 papers discussing the usage of visual stimuli delivered by AR/MR or similar technology to enhance the performance of physical exercises in medical rehabilitation. The taxonomy bridges the gap between a medical perspective (Patient Type, Medical Purpose) and the Interaction Design, focusing on Output Technologies and Visual Guidance. Most approaches aim to improve autonomy in the absence of a therapist and increase motivation to improve adherence. Technology is still focused on screen-based approaches, while the deeper analysis of Visual Guidance revealed 13 distinct, reoccurring abstract types of elements. Based on the analysis, implications and research opportunities are presented to guide future work.

## 1. Introduction

As people age, serious illnesses and injuries requiring medical rehabilitation become more common [1]. The demographic shift towards a higher mean age and life expectancy in many industrial nations means that the demand for rehabilitation increases every year. Types of rehabilitation include medical, vocational and residential/community rehabilitation. While vocational rehabilitation focuses on enabling persons to return to, or remain in employment, Residential rehabilitation services offer group living settings to disabled people to enable them to live as independently as possible; medical rehabilitation aims at minimizing consequences of disease, illness, injury, aging, and congenital factors. As we focus on the use of augmented and mixed reality in physical exercises, the area of medical rehabilitation is most relevant to our research. It consists of a variety of treatment methods. Among others, these include the fields of physiotherapy, occupational therapy, and physical therapy [2]. These three types of therapy are very close to each other. Therefore, we will refer to all types of physical exercises performed by patients within these fields by the umbrella term *physical rehabilitation*. The three kinds of therapy are mostly used for similar clinical pictures, such as physical damage and disabilities due to illness or accidents. The spectrum ranges from damage to the spine and joints to diseases of the central nervous system or the effects of a stroke. Expanding on the latter, according to the World Health Organization (WHO), strokes are not only often fatal but also the third leading cause of disability worldwide [3], reducing the mobility in half of the stroke survivors aged 65 and above [4]. Patients having suffered from a stroke often have long-term impairments that restrict them from performing activities of daily life such as work, leisure, or sports [5], requiring a rehabilitation process to recover from illness and regain a healthy lifestyle, specifically improving mobility, coordination, strength, and endurance. Besides strokes, many other health conditions, injuries, and disabilities require intensive rehabilitation programs which are constructed similarly and share common challenges, such as the active performance of physical exercises, tasks, or activities by the patient [6,7,8]. Therefore, in this paper, no focus will be put on a specific condition nor a type of therapy, but physical rehabilitation processes in general.

Conventionally, specialized prevention and rehabilitation facilities provide in-clinic physical therapy which is quite demanding in terms of time and cost, requiring supervision as well as individual treatment plans and modifications as per the patient’s progress [2]. Simultaneously, the number of physical therapists employed at these facilities has been reduced over the last decade [1]. Within both in- and outpatient rehabilitation facilities, a single therapist commonly supervises multiple patients simultaneously, which makes it quite difficult for them to support each patient adequately. Considering the high rehabilitation costs and the limited availability of physical therapists, patients often do not receive the close supervision needed. Further, rehabilitation programs take place not only in specialized facilities but also require repetitive exercising at home for several months, if possible, without supervision by a therapist [2]. This repetition makes the process quite lengthy and boring. Consequently, patients often lose motivation to continue the program. Additionally, the current COVID-19 (a highly infectious respiratory disease) pandemic has caused restrictions on outdoor mobility and gatherings, including therapy sessions, leading to a decline in overall activity [9], especially among older people. This further emphasizes the importance of exercises to be performed at home, requiring less or remote supervision by a therapist.

A vast amount of research has aimed to employ and adapt technology to support the rehabilitation process and overcome these problems. In particular, different implementations of Augmented and Mixed Reality (AR/MR) technologies have been applied and, as we will outline in more detail in this paper, these show that such approaches might indeed help to support the rehabilitation process. The advantage of AR/MR is that it allows merging visual digital cues with the real environment of the patient, thus letting the user focus on the physical task or exercise while still providing visual guidance in a digital form which is anchored to the real world to provide context. In their systematic literature review, Cavalcanti et al. approach the relationship between Augmented Reality and motor rehabilitation from a different angle [10]. They analyze the existing literature to provide an overview of the different types of assessment methods to evaluate the usability of such technologies. While not the primary focus, they also give an overview of affected body parts and medical conditions that were addressed in the past. However, their review does not detail the medical purpose nor interaction design of the individual approaches.

The current literature, as will be outlined in examples later, sheds light on significant aspects of visual elements that have helped patients to benefit more in the rehabilitation process utilizing this technology and can be investigated further. For example, different forms of instructions or feedback delivered by visual cues (text, image, 3D visualization) and sometimes accompanied by audio can be utilized to communicate the correct movements and thereby enable the patient to perform the exercises correctly [11]. Besides that, SleeveAR [12], an interesting novel approach, provides AR projection-based real-time performance feedback that can assist the patients to perform exercises autonomously, without direct supervision by a therapist. The work by Garcia et al. [13] uses mobile AR and game-based approaches to treat people suffering from ankle sprains. The game-based approach offers interesting visual elements that increase the patient’s motivation and adherence to continue the rehabilitation program. AR/MR systems such as [14,15] offer interesting visualizations that can prove to be more effective than traditional therapy approaches.

Due to these potentially beneficial properties of AR/MR in the context of physical rehabilitation and the vast amount of existing work in this area, we identified the need for a comprehensive literature review. From the perspective of HCI, it also seems necessary to address the in-depth examination of visual guidance that can benefit HCI researchers or practitioners in the long run. The categorization will not only help to provide a better understanding of the visual guidance elements used in the rehab applications but will also uncover interesting insights and identify research gaps. Thus, this paper aims at providing a taxonomy which:

- Identifies different forms of *Visual Guidance* aimed to help patients in their rehabilitation, for example, by allowing them to perform exercises autonomously at home without any supervision by a therapist;
- Provides an overview of the current use of the variety of *AR/MR Technologies* used for visual output in the field of physical rehabilitation;
- Derives insights regarding the relations between *Patient Types*, *Medical Purposes*, *Technologies*, and specific *Visual Guidance* approaches used to support the patient in their rehabilitation process.

## 2. Materials and Methods

To gain a thorough understanding of the different AR/MR visual guidances that assist patients in the rehabilitation process, the relevant literature was retrieved from different databases, containing a mixture of medical and HCI literature. For the chosen research question, we decided to perform the literature review in the form of a scoping review. Unlike systematic reviews, scoping reviews aim to address broader topics and sources employing different methods and study designs, while focusing less on narrow questions nor the assessment of quality. They are most useful for providing an overview of the extent, range, and nature of currently evolving areas of research, to summarize research findings or to identify research gaps [16,17]. In our review, we aim to thoroughly explore these aspects with a focus on research on augmented and mixed reality applications to support physical exercises in medical rehabilitation. Therefore we consider a scoping review to be in our case the appropriate method for the purpose of developing a taxonomy.

The analysis process was initialized with a prescreening focusing on the abstract of a paper, followed by a full-text assessment for a relevant subset. On the resulting set of references, we applied an open-coding scheme, meaning that relevant categories and codes were not predefined but identified based on the screening results and adjusted through the thorough analysis of the reviewed studies. This way, a meaningful segmentation could be derived in relation to the research question. Three main and eight subcategories evolved during the coding process and were subsequently applied to the whole data-set. Codes within a category were not mutually exclusive. The classification of the final set of papers along the coding scheme was carried out by the first three coauthors of this review. As with the selection process, each application was reviewed by two researchers. Discrepancies were resolved in discussion with the third reviewer. A detailed description regarding the eligibility criteria and study selection process is explained below.

### 2.1. Eligibility Criteria

As motivated in the introduction, our main focus is on the following items:

- The performance of physical exercises in medical rehabilitation. As mentioned above in the introduction, medical rehabilitation consists of a variety of treatment methods, including the fields of physiotherapy, occupational therapy, and physical therapy [2]. As these types of therapy are interrelated to each other, we will refer to all kinds of physical exercises performed by patients within these fields by the term *physical rehabilitation*.
- The output technologies Augmented and Mixed Reality (AR/MR) as means to support the different types of physical exercises. The available vast amount of research in combination with the potential that future developments of the technology offer, we think, merits such a rather strict technological focus of this literature review. As has been the approach by other researchers (e.g., [18]), we will not distinguish between AR and MR but use the terms interchangeably or refer to them as AR/MR. As a foundation, we mostly follow the definition of Azuma, who summarized in his early survey of 1997 that AR (a) combines the real and virtual, (b) is interactive in real-time, and (c) is registered in 3D [19]. In particular, the last requirement is not always met with all types of output technology. For example, smart glasses often superimpose a digital layer without registering this in 3D. For the matter of thoroughness and because the practical definitions of AR and MR are also evolving [20], we still included such approaches in our review. It is noteworthy to mention that we did not actively exclude applications employing Virtual Reality (VR) as long as our eligibility criteria were met. However, the number of VR applications found may not be representative, since we did not explicitly search for the term Virtual Reality during our paper selection process.
- The actual use of visual stimuli or visual guidance to support patients during the rehabilitation task. Here, we did not limit our review to graphical stimuli but included representations with text as well.

### 2.2. Literature Sources and Search Strategies

We based our review on the following five databases: Web of Science, ScienceDirect, PubMed, IEEE Xplore, and ACM. This diverse focus is necessary considering the multidisciplinary nature of the research question, connecting information technology, especially the area of human–computer interaction, with the field of medical rehabilitation. Web of Science and ScienceDirect offers such a broad, multidisciplinary overview of the scientific spectrum. PubMed is the leading database within the medical field, including research on physical therapy from both the patients’ and therapists’ viewpoints. IEEE Xplore and ACM, on the other hand provide a stronger technological perspective.

We chose search terms in accordance with the research question and iteratively expanded them based on the keywords of relevant references found. To be shortlisted in the database search, the following two requirements had to be met:

- The research must take place in the context of physical rehabilitation, i.e., physical exercises in medical rehabilitation. Therefore, at least one of the following search terms had to be included in the title, keywords, or abstract: “*Physical Therapy*”, “*Physiotherapy*”, “*Physical exercise*”, “*Physical rehabilitation*”, “*Occupational Therapy*”, “*Stroke rehabilitation*”.
- Furthermore, the research must use some kind of AR-/MR-based visual guidance or stimuli to support the patient in their rehabilitation. Consequently, one of these search terms must be found in the title, keywords, or abstract in addition to the first requirement: “*Mixed Reality*”, “*Extended Reality*”, “*Augmented Reality*”, “*Visual Cues*”, “*Visualization*”, “*Visualisation*”, “*Video-based*”.

Early database searches in the field of medical rehabilitation emphasized the importance of research aimed at the rehabilitation of stroke patients, resulting in an explicit mention of this condition in the keywords. In addition, the search did not require AR/MR terms but also allowed papers to be examined which used some kind of visual guidance.

We included original articles, conference papers, and demonstrations in the search, as well as literature reviews, as these may reference interesting papers otherwise not found in the search. In order to illustrate both the progression and the state of the art of research in the highlighted area, no restrictions were placed on publication year. Due to the terms chosen, the focus of the search was on English publications but encountered references in other languages were translated and considered as well. The last search took place on the 27 July 2021.

### 2.3. Study Selection

**Figure 1:**
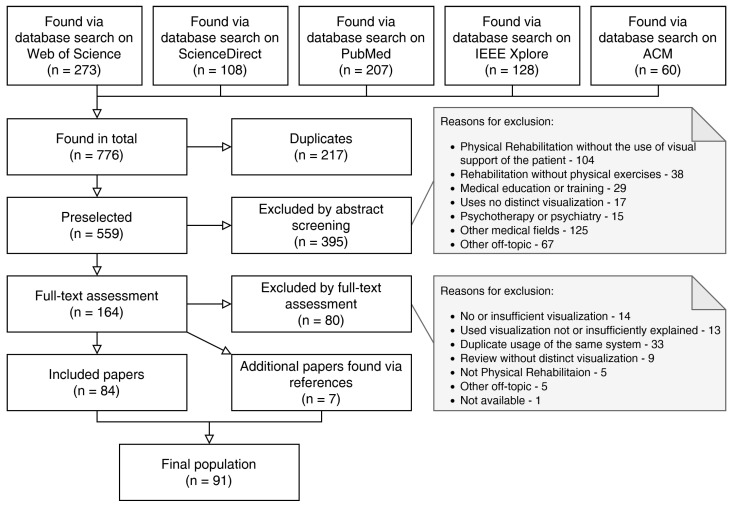
Flow chart of the study selection process, own representation based on [16].

A total of 776 references were found. An overview of the selection process is depicted in Figure 1 and summarized below. First, 217 duplicates were removed. The abstracts of the remaining 559 papers were each screened independently by two different researchers. Discrepancies were resolved by discussion with a third researcher, who would make the final call. Another 395 references were excluded in this step in accordance with the eligibility criteria due to the following reasons:

- The reference addresses physical exercises in medical rehabilitation, but without the use of AR/MR-based or comparable visual guidance or support for the patient (104).
- The reference addresses medical rehabilitation but not physical exercise (38).
- The reference addresses the use of visual support in medical education or training, aimed at the medical professional (29).
- The reference uses no distinct visualization, but instead either considers the feasibility of conventional rehabilitation delivered via video calls or the effect of commercially available mobile games on general activity (17).
- The reference explicitly considers psychotherapy or psychiatry without physical exercise (15).
- The reference addresses other medical fields, such as medical imaging or surgery (125).
- The reference is off-topic in other ways, for example, including other data visualization in Machine Learning, Artificial Intelligence, Veterinary Sciences, applications not employing AR/MR or VR, and game reviews (67).

This resulted in 164 references which were assessed in more detail, i.e., via a full-text analysis, employing the same dual control principle mentioned before. Another 80 references were excluded in this step for the reasons stated below:

- The references utilize no or an insufficient level of visualization (14).
- The references mention a system using visualization but do not explain it in enough detail, which would help other researchers and practitioners to build upon their results (13).
- The references utilize a system or application which was already (and better) explained in another paper included in the data-set. This took place many times as research groups would commonly deploy a single application for a number of distinct papers, such as a preliminary report, a feasibility study, a paper explaining the technical development, and a clinical long-term study (33).
- The references are literature reviews showing no distinct visualization, but refer to other potentially interesting papers (9).
- The references are off-topic in other ways (5).
- In addition, one single reference could not be procured (1).

This assessment left 84 studies to be included in the review. Despite our focus on AR/MR technology, we did not exclude papers employing Virtual Reality, as long as they fit our search terms, explored the use of visualization to support patients within the field of physical rehabilitation, and sufficiently explained their visualization. Additionally to the initial search, we screened the references cited by the set of 164 in-depth assessed papers and identified 7 further references which we included as well. This led to a final set of 91 references which were examined in the following. The publication years of the considered papers range from 2002 to current releases.

## 3. Results

### 3.1. Taxonomy Construction

**Figure 2:**
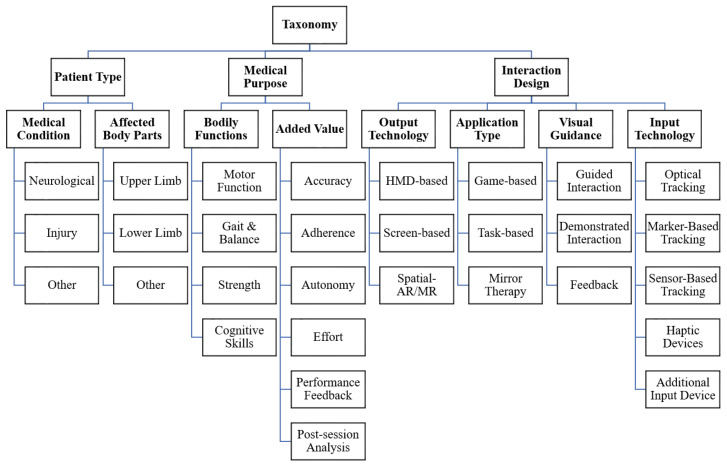
Overview of the Taxonomy.

Among the 91 references, some include two or more diverse approaches or applications, such as for means of comparison, for distinct tasks of different body parts, or to allow the user to choose out of a set. In cases where there was a clear distinction between applications, all were considered individually in our review. However, very similar applications, such as slightly modified pick-and-place-tasks, within a single reference were treated as one. As a result, we identified 114 distinct applications, which form the basis of our taxonomy. An overview of the 114 applications, structured by reference and the coding scheme of the taxonomy, is provided in the Appendix A. Overall, we were able to identify three main categories and eight sub-categories, which form the basic hierarchical structure of the taxonomy (see Figure 2). Other items within sub-categories were chosen in case of the larger number of matching applications and distinct features that could further be identified.

Firstly, we identified as the main category, that the 114 applications focused on different **Patient Types**, which could further be separated in different **Medical Conditions** as well as **Affected Body Parts**. While these may not be mutually exclusive from a medical point of view, not all references provided the necessary medical details and rigor, which made us opt for such a rather practical separation.

The most prevalent **Medical Conditions** considered in the references were of *Neurological* nature (78%, 89 applications), predominantly *Stroke* (73%, 83 applications) with only few mentions of other conditions such as *Parkinson* (3%, 3 applications). Further, interventions following *Injury or Surgery* (9%, 10 applications) were considered as well. Finally, a set of *Other* (25%, 28 applications) with either unspecific or diverse conditions was identified. For example, we found a single study examining neck pain [21] as well as an article focusing on the technical motion-capturing aspect of a proposed full-body physical therapy system, without further specifying any medical condition [22].

Regarding the **Affected Body Parts**, 81 (71%) of the applications especially aimed at *Upper Limb*, out of which 52 (46%) were focused on the *Hands*, *Fingers*, or *Wrist*, 20 (18%) on the *Shoulder*, and 21 (18%) on the *Elbow*. A total of 33 (29%) applications considered the *Lower Limb*, while only 4 (4%) of these further emphasized either the *Knee* or *Ankle*. Another three (3%) applications regarded *Other* parts of the body, namely the neck once [21], and movement of the body in general twice [23,24].

Secondly, we identified the main category **Medical Purpose**, covering the reasoning behind the different applications from a medical perspective. We further subdivided this category into **Bodily Functions** and **Added Value**.

The **Bodily Function** describes the primary, functional purpose of the physical rehabilitation. Four types of functions were identified. In total, 96 (84%) of the applications were aimed at restoring or improving *Motor Functions* of certain joints or limbs. A further 26 (23%) aimed to improve *Gait or Balance*, while 24 (21%) aimed at building *Strength*. Only 13 (11%) applications explicitly addressed the enhancement of *Cognitive Skills*.

The **Added Value** captures the benefits that researchers aim to achieve through the means of technology used in the applications, and thereby go beyond what conventional physical therapy provides. Most of the applications aim to provide several such benefits. The most common added value is an increase in *Autonomy*, allowing patients to perform the exercise without close supervision by a therapist, which applies to 91 (80%) of the applications. An additional goal is to overcome the repetitive and boring nature of many therapeutic exercises by increasing the motivation of patients. Here, two aspects play a role. First, 87 (76%) applications aim to improve patients’ *Adherence*, meaning their likeliness to continue with their rehabilitation program over a prolonged period, while 47 (41%) aim at boosting their *Effort* during a session. Motivational factor is considered by 75 (66%) of applications, which assist the patient during an exercise by providing real-time *Performance Feedback*, whereas 46 (40%) allow for an *Analysis* of the performance after the session. Among all, a noteworthy added value, which a technological solution may provide, is an increase in patients’ *Accuracy* to perform the exercises, which is considered by 39 (34%) of the identified applications.

The third and the last main category, **Interaction Design**, revolves around the technical solutions as implemented in the different applications. It, therefore, comprises the used **Output Technology**, the **Application Type**, the concrete kind of **Visual Guidance** employed to aid the patient, as well as the **Input Technology** required for the AR/MR technology to work.

As for the **Output Technology**, 72 (63%) of the applications could be considered *screen-based*, i.e., making use of a type of 2D display, mobile phone, or projector image to superimpose digital content on video-based real-world imagery. Only 8 (7%) of these applied a handheld *Mobile Phone-based AR/MR* approach, similar to Pokémon Go and the like. Another 30 (26%) of the applications used a Head-Mounted Display (HMD), out of which 13 (11%) used a *VR HMD* and 17 (15%) an *AR/MR HMD*. Another 14 (12%) of the applications delivered the application via a *Spatial-AR/MR* approach, which uses a projector to display digital content onto the patient and his surroundings, thereby registering the virtual content in 2D space [25].

The **Application Type** can be either *Game-based*, signifying playful and fun solutions employing gamification elements, as 55 (48%) of the applications were, or *Task-based*, as were 53 (46%). Compared to their game-based counterpart, *Task-based* applications emphasize a more serious approach, focusing on the execution of simple, repetitive exercises rather than motivational aspects. As another item in this category, we extracted *Mirror Therapy* as a highly specific type found in 12 (11%) of the applications. In mirror therapy, the brain is tricked into thinking that an affected limb, for example, an amputated arm, is able to move without pain. This is achieved through putting a mirror in a specific spatial configuration next to the healthy arm. The brain now connects the mirrored image with the affected limb, so that when the patient tries to move both arms (and only the healthy fully responds), it assumes that also the affected limb is moving correctly, helping to train the relevant function and mitigate pain [26].

The main emphasis of our analysis lays in the identification of the **Visual Guidance** elements. In total, 13 reoccurring abstract concepts were found here, which we classified into three groups: **Guided Interaction**, **Demonstrated Interaction**, and **Feedback**. The concepts are explained in the following.

**Figure 3:**
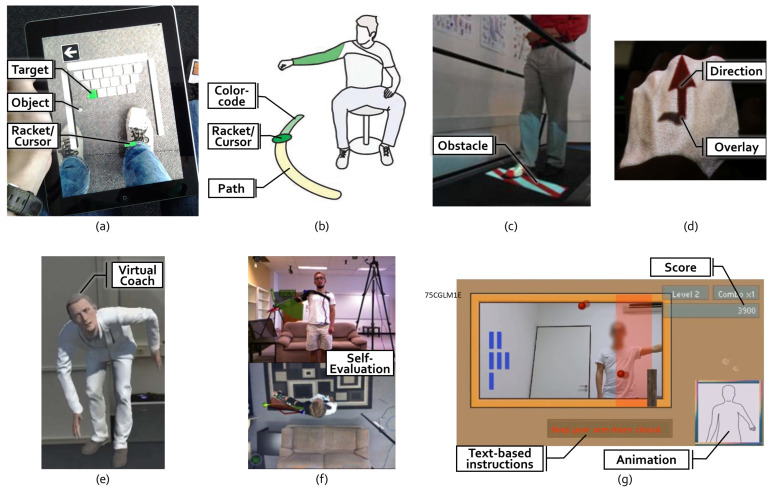
Illustrative examples of visual guidance within the selected references: (**a**) game-based ankle exercise using a racket to direct an object into a target [13]; (**b**) task-based elbow and shoulder exercise guiding the patient’s cursor along with a color-coded path [12]; (**c**) task-based gait training on treadmill displaying an obstacle to be avoided by the patient [35]; (**d**) arrow overlay projected onto the patient’s hand guiding him in the right direction [30]; (**e**) virtual coach demonstrates the correct performance of an exercise [37]; (**f**) alternative angle displayed on the screen in front of the patient allows him to evaluate his movements [28]; (**g**) game screen extended with text-based instructions, score, and animation demonstrating the correct performance of the exercise [11].

The group **Guided Interaction** includes such visual support elements, aiding the patient by demanding immediate interplay. We identified the following elements:

- **Target:** A total of 64 (56%) of the applications use some form of *Target*, which we define as a spatial destination to be reached by the patient. For example, in Garcia and Navarro [13], the user controls a ball to hit bricks that act as a target with a paddle controlled by the foot movement (see Figure 3a). Similarly, another game in Bouteraa et al. [27] shows randomly appearing targets that patients need to grasp with their hand and the help of an exoskeleton.
- **Path:** Related to targets are *Paths*, which not only show the destination but also a trajectory to be followed. Compared to targets, paths provide information during the entire execution of the exercise. In total, 21 (18%) of the applications use this type of visualization. In SleeveAR [12], a path is shown to direct the arm movement in order to execute the exercise (see Figure 3b). Physio@Home [28] visualizes a wedge that includes a movement arc for proper arm guidance (see Figure 3f).
- **Direction:** Overall, 15 (13%) applications do not show an entire path but indicate a *Direction* to be followed instead, usually visualized by an arrow. In the ARDance game [29], the user has to move the AR marker towards the left or right diagonal directions to complete dance moves. LightGuide [30] uses a simple 3D arrow projected onto the user’s hand to visualize the direction of movement. It also uses the Hue cue, a visual hint that uses spatial coloring to indicate direction (see Figure 3d). Tang et al. [28] also visualize such an arrow in addition to their path (see Figure 3f).
- **Object:** The most common element is *Objects*, found in 69 (61%) of the analyzed applications. Objects, unlike targets, can be interacted with in some way. For example, in the Ocean Catch Game by Park et al. [31], the user trains grasping movements by catching virtual fish. Similarly, Alamri et al. created a Shelf Exercise [32], which reenacts the motion of placement by showing a virtual cup that can be placed on different spots in a shelf by the user.
- **Racket/Cursor:** A *Racket or Cursor* is an object directly controlled by the patient which allows them to manipulate other objects; 21 (18%) of the analyzed solutions utilize such a racket. For example, with the help of a marker, the bar is used to hit the ball in a game of Pong as shown by Dinevan et al. [33]. FruitNinja as applied by Seyedebrahimi et al. [34] shows a circle to depict the current position of the hand to control fruit targets appearing on the screen.
- **Obstacle:***Obstacles* describes hindrances to be avoided or bypassed. They are employed in 11 (10%) of the applications. To facilitate practicing gait and balance exercises, the obstacles can be projected directly onto the treadmill [35] (see Figure 3c) or shown in the form of virtual objects such as blocks or tree trunks [36] to perform certain leg movements.

*Visual Guidance* can also be provided by **Demonstrated Interaction** of the correct execution of the exercise. Demonstrations can be delivered in the ways mentioned below.

- **Recording:** The demonstration may be delivered by a prerecorded *Video, Animation*, or *Picture*, as it was the case in 23 (20%) of the analyzed applications. For instance, UNICARE Home+ by Yeo et al. [38] shows a guide video on the side of the screen to depict exercise movement. Another example from Khan et al. [39] uses the “follow the leader” approach by showing an animated virtual avatar to be followed by the user.
- **Virtual Coach:** Unlike with an unchanging recording, a *Virtual Coach* provides interaction with the user. Such a coach was used in seven (6%) of the analyzed solutions. The holographic representation of a virtual coach as depicted in Mostajeran et al. [37] allows users to look at the coach from different angles, see instructions from the same perspective and fosters interaction (see Figure 3e).
- **Overlay:** An *Overlay* displays a visual instruction directly perceived on the user’s body. In total, 15 (13%) applications employed such an *Overlay*. In a Kinect-based system as shown by Pachoulakis et al. [40], the user’s body is overlaid with skeleton joints displaying the trainer’s movement, allowing him to follow the exercise.

The last group of visual guidance contains elements providing **Feedback**. These elements guide the patient indirectly by adding another layer of information related to the task or exercise. Often, these elements inform the patient about the quality of their performance.

- **Text:** In total, 23 (20%) applications deliver *Text-Based Instructions* or *Feedback*. ARKanoidAR [11] uses text-based instructions to guide the user in performing the exercise correctly (see Figure 3g). Additionally, a virtual piano game [41] motivates patients by showing text-based feedback such as “Well Done” based on the performance of the user.
- **Score/Graph:** A total of 60 (53%) of the analyzed solutions provide the patients with some kind of informative *Graph, Score or Progress Bar* related to their performance. When the task is performed correctly, the score is increased, as depicted in FruitNinja [34] or ARKanoidAR [11] games. Such game elements increase the user’s motivation to keep performing the exercise.
- **Color-code:** Information can also be transported using a *Color-Code* which is used by 23 (20%) applications. To illustrate if the user has reached the target appropriately, interACTION [42] uses a color-coded mechanism to inform the patient. The Target is originally displayed in red. Once the user approaches the target, it changes its color to yellow and once the target is reached, it turns green. Another approach used by [15] is to compare the user’s pose and the desired pose with a color code. Once the user’s pose matches the desired pose, the circular glyphs are highlighted in green.
- **Self-Evaluation:** Altogether, 26 (23%) applications grant the patient some kind of improved *Self-Evaluation* delivered by either another camera angle or mirror to visualize the real affected body part better as shown in Physio@Home [28] (see Figure 3f), or by displaying an avatar or skeleton based on motion tracking data to grant information on the patient’s own movement. One such example is depicted in [26] for gait symmetry, where the user’s whole body movement is shown via an avatar with different views for a better understanding of gait deviations.

Lastly, the **Input Technology** utilized to allow interaction between the patient and system is considered as part of the *Interaction Design* category. Most applications require some sort of motion capture with the most common method being *Optical Motion Tracking*, employed by 48 (43%) applications. Examples include the work of [40,43] that is based on Kinect motion tracking, and games for hand rehabilitation that employ Leap motion tracking [44]. Moreover, quite popular is the use of *Marker-based Motion Tracking*, which is utilized by 38 (34%) solutions. Few prototypes such as Physio@Home [28] and gait symmetry application by [26] used Vicon motion tracking cameras for precise tracking. Vicon tracking system requires wearing explicit markers on different body parts. Another 19 (17%) depend on nonoptical *Sensor-based Motion Tracking* such as Xsens used by [36]. Nine (8%) applications used some form of *Haptic Device* to simulate tactile sensation in AR environment when users touch virtual objects as used by [45] and 29 (26%) employed an *Additional Device*, such as a treadmill used by [46] for gait rehabilitation.

### 3.2. Time-Based Analysis

**Figure 4:**
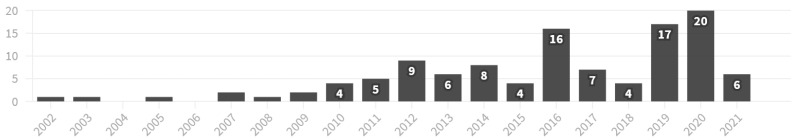
Distribution of the selected references by publication year.

**Figure 5:**
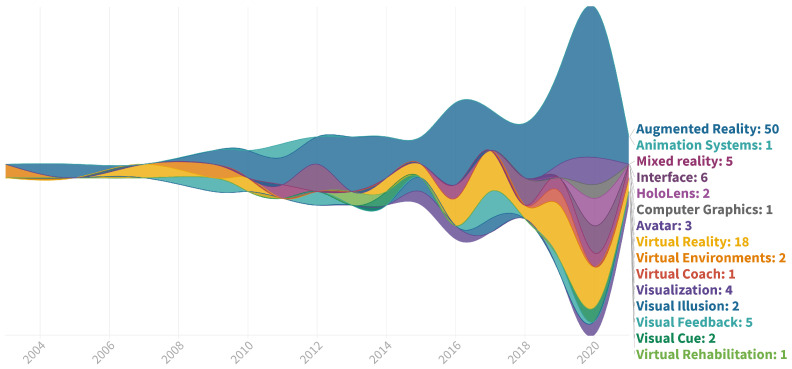
Usage of visualization-related keywords in the selected references.

We identified 91 references published between 2002 and the first half of 2021 related to the field of AR/MR visual guidance in physical rehabilitation. Only 8 of these were published prior to 2010 followed by a significant increase in research interest, peaking in 20 publications in the year 2020 alone. The detailed distribution per year is illustrated in Figure 4. The most commonly used keywords provided by this set of references are displayed in Figure 5. The data was harmonized in this step to group different representations, such as Mixed-Reality and Mixed Reality, and plural forms of the words. A significant bias towards the use of the term *Augmented Reality* across the entire time period is apparent, outnumbering *Mixed Reality* 10-fold. *Virtual Reality* was mentioned in 18 references despite not being included in the search terms. In contrast, seemingly general terms such as *Visual Cues*, *Visualization*, and *Visual Feedback* find much less use.

### 3.3. Patient Type and Bodily Functions

As became apparent during the open coding process, a large majority (73%) of the identified references explicitly focus on rehabilitation following *Stroke*. We did not expect such a prevalence as our search encompassed five other, broader search terms as well. In contrast, rehabilitation following *Injuries or Surgery* is only explicitly the focus of 10 (9%) of the references. Similarly yet less pronounced is the accentuated focus of the *Upper Limbs* compared to the *Lower Limb*, with 81 (71%) compared to 33 (29%) references. Distinct differences become apparent between research in these areas. These findings coincide with those of Cavalanti et al. who, in their literature review of studies employing AR in rehabilitation, found a strong prevalence of *Upper Limb* research and a severe yet less pronounced focus on *Stroke* [10].

**Figure 6:**
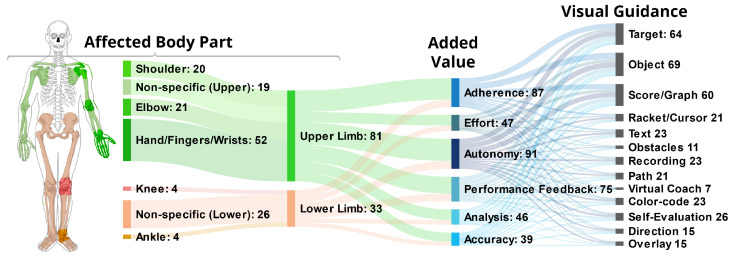
Connections between selected elements of the reviewed literature.

**Figure 7:**
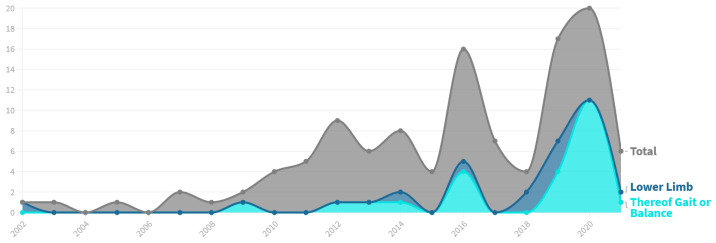
Share of research aimed at *Lower Limb*.

- First, studies aimed at *Upper Limb* are predominately focusing on individual parts of the limb, sometimes multiple, as seen in Figure 6. Among the 81 references covering *Upper Limb*, 51 (63%) target either the *Hand, Fingers or Wrist*, 21 (26%) the *Elbow* and 20 (25%) the *Shoulder*, leaving only 19 (23%) references without a specific focus. In contrast, the 33 references regarding *Lower Limb* are overwhelmingly nonspecific (79%) with only 7 applications (21%) focusing on either the *Knee*, *Ankle* or both.
- Further, there is a difference in the targeted *Bodily Functions* depending on the considered *Body Part*. 79 (98%) out of the 81 *Upper Body* applications target the restoration or improvement of specific *Motor Functions*, while 19 (23%) aim to increase the patient’s muscle *Strength*. In comparison, with 24 (73%) the largest fraction of the 33 *Lower Limb* research addresses the patient’s *Gait or Balance*, especially in order to prevent future falls. Improvement of *Specific Motor Function*, however, is only targeted in 19 (58%) references here. Muscle *Strength* is also considered less often, finding explicit mentioning in five (15%) of the references.
- Lastly, the research focused on both *Lower Limb* and *Gait* has increased immensely in recent years, as illustrated in Figure 7. Prior to 2019, we identified a total of 13 references targeting *Lower Limb*, representing only 18% of the research papers published in this time frame. Further, only eight of these (62%) aim at *Gait or Balance*. Compared to that, 20 out of 43 applications published since 2019 target *Lower Limb* (47%). Additionally, the share of *Lower Limb* research focusing *Gait or Balance* rose to 80%.

### 3.4. Medical Purpose and Application Types

Differences become apparent between the variants of *Application Type* and their relation with *Medical Purpose*:

- Regarding the *Bodily Functions*, 11 out of the 13 (85%) applications aiming to provide *Cognitive* training do so by employing *Game* elements.
- The 55 *Game-based* applications are especially often used to increase the patients’ motivation, with 51 (93%) of them targeting increased *Adherence*. For the 53 *Task-based* applications, *Adherence* is a much less prominent goal, which is only addressed by 31 (58%) applications. In total, 30 (55%) of the *Game-based* applications further aim to increase the patients’ *Effort* during the session, compared to only 16 (30%) within the set of Task-based approaches.
- Instead, 28 (53%) *Task-based* applications focus on increased *Accuracy*, compared to only 11 (20%) within the set of *Game-based* applications.

**Figure 8:**
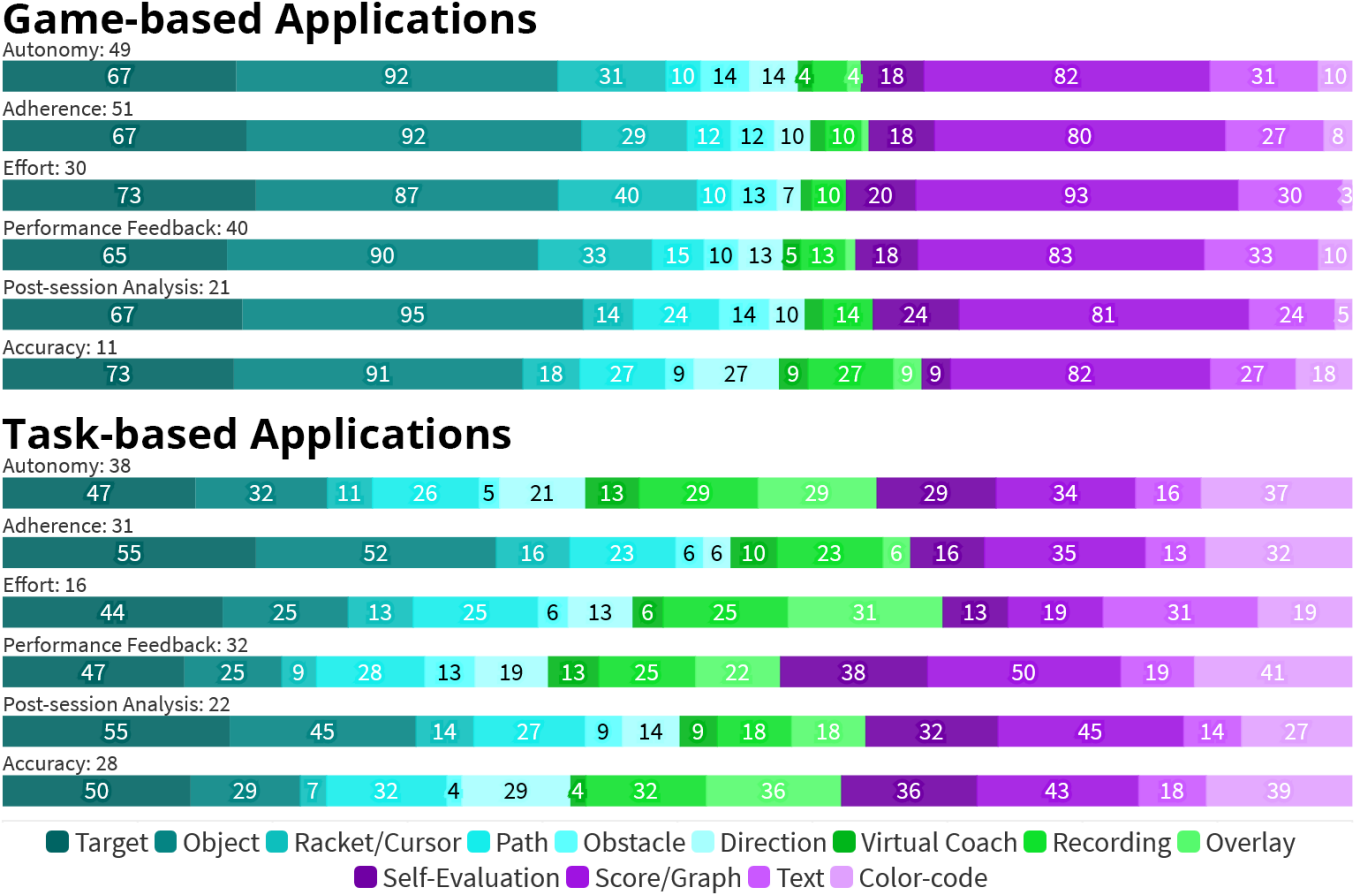
Usage of *Visual Guidance* elements by *Application Type* and targeted *Advantage*. Numbers on the bars represent the percentage of applications utilizing a *Visual Guidance* element. The bar’s width represents the relative frequency within the respective *Added Value*.

Figure 8 shows the usage of *Visual Guidance* elements by *Application Type* and targeted *Added Value*. The main insights are summarized below.

- In general, *Task-based* applications are more balanced in their use of visual elements, while *Game-based* are more focused on certain elements, namely *Objects* (91%), *Scores or Graphs* (80%), and *Targets* (65%).
- The most common visual elements implemented in *Game-based* applications are from the *Guided Interaction* group. Elements from this group are used in 53 *Game-based* applications (96%) and make up 59% of all elements used here. In comparison, they are used in 38 *Task-based* applications (72%) and account for 46% of elements used.
- In contrast, only nine *Game-based* applications (16%) utilize one form of *Demonstrated Interaction* each, accounting for 4% of elements. Within *Task-based* applications, 24 (47%) employ at least one *Demonstrated Interaction* element, accounting for 19% of all elements.

## 4. Discussion

In this section, we will take a closer look into the *Interaction Design* approaches and in particular discuss different approaches and implications within the categories *Visual Guidance* and *Output Technology*.

### 4.1. Implications for Visual Guidance

Garcia and Navarro were able to show that the MobileReh [13] app enhanced overall user interaction for ankle sprain rehabilitation by providing several *Guided Interaction* elements, such as *Targets*, *Objects*, *Paths*, and *Racket/Cursor*. The game required no controllers or sensors to operate. The main challenge the authors reported was to keep the game engaging, which they tried to tackle by providing higher difficulty levels. Alamri et al. found that virtual *Objects*, such as a cup in their Shelf Exercise approach [32], tend to be motivating for users and help them to keep the exercises engaging. The simple activities of daily life such as pick-and-place movement can be made more interesting if real-world objects could be overlaid with virtual objects. The seamless integration of virtual objects in the real environment can be achieved by advanced AR/MR technology, such as *HMD-AR/MR* or *Spatial-AR/MR* and the authors conclude that these can enhance the user experience along with increasing patients’ participation in the rehabilitation program. For applications aiming to improve gait adaptability, Fonteyn et al. and subsequently Timmermans et al. were able to show that *Guided Interaction* involving the element of *Obstacles*, i.e., avoiding/dodging hindrances, can indeed improve gait performance [35,47].

Seyedebrahimi et al. conducted a study to compare different implementations of the FruitNinja game [34]. They compared what they call an AR and VR mode. Given the description in their work, however, we regard these two variants as referring to the *Spatial-AR* and *Screen-based* category, respectively. The authors observed that the *Spatial-AR* mode provided better hand-eye coordination which plays a significant role in completing tasks. The *Objects* were projected directly on the tabletop that made it easier for users to interact. Compared to this, in the *Screen-based* mode, where the output was displayed on a monitor, visual and proprioceptive feedback were uncoupled because the gaze was directed on the monitor and not their hand, resulting in poor motor performance.

**Guided Interaction vs. Demonstration and Virtual Coach**: Several researchers compared their *Guided Interaction* approaches to *Video Demonstration*. For example, Sousa et al. found that the movement *Path* as shown in SleeveAR [12] showed better results when performing the exercise as compared to a *Video Demonstration* alone. The *Path* not only guided the user on how to move but drawing the trajectories over the original exercises helped understanding the areas of improvement. In a similar manner, Tang et al. performed an experiment to investigate if the wedge visualization (including a movement arc and a *Directional* Arrow), as shown in Physio@Home [28], helped users to perform the exercise better than *Video Demonstration*. It was shown that the movement *Path* guided users better to perform the steps correctly for hand exercise. However, for elbow exercise, the results were not any better than *Video Demonstration* due to a technical limitation. Overall, the corrective guides helped people to realign themselves when needed. In their work, Sodhi et al. explored on-body projected hints (*Overlay*). In their approach LightGuide [30], this helped users to perform the movement more accurately compared to a simple *Video Demonstration*. It was reported that *Video Demonstration* is unable to adequately show next steps. In comparison, visual hints such as a 3D *Arrow* helped users to exercise at their own tempo and guided them to perform the movement in a particular direction. 3D Pathlet on the other hand served as a feedforward component to guide the user in advance what is ahead in the *Path*. The question is still open if such visual hints on a *Screen* will allow users to perform the movement as accurately as when they are projected on to the body.

While these comparisons with *Demonstrated Interaction* mostly chose *Video Recordings*, other researchers have explored *Virtual Coaches* as a digital representation or surrogate for a real therapist. Mostejarean et al. [48] conducted a study to find out if *Virtual Coaches* in the form of holographic representations have potential to help older adults in *Balance* training. Results suggest that a *Virtual Coach* was well perceived by the users and the holographic representation of the *Virtual Coach* made it easier to look at it from different perspectives-something that is difficult to achieve with *Screen-based* systems. The *Virtual Coach* was able to interact with users with the help of audio and speech feedback. Users reported that the visual representation of the avatar should be more human-like to make it feel real and alive. The authors conclude that such a personalized coaching system has the potential to be used in home-based therapy setting. Another form of *Virtual Coach* was used by Han et al. [49]. They *Overlaid* the *Virtual Coach*’s guided bone structure onto the user’s body to perform Tai-Chi maneuvers recorded by a motion capture system. The participants reported that they were able to perform tasks well using this immersive technique and better compared to simple instructions by pictures or words.

**Feedback**: Another approach to address gait performance was investigated, for example, by Liu et al, focusing on avatar-based feedback [26]. They found that such an approach provides many advantages as it helps to *Self-Evaluate* the movements of the user. They showed that the availability of multiple views of an avatar allows different perceptions, depending on the current users’ need which makes it quite versatile in nature. Additionally, it also provided unique concurrent information on walking performance and was able to show improvements compared to communicating such information directly by clinicians or therapists. Bell et al. evaluated the real-time visual *Feedback* in their interACTION app, which was well perceived by users in a study conducted to check the feasibility of the system [42]. It was reported that the visual *Feedback* helped users to perform the exercises correctly. The real-time qualitative and quantitative *Feedback* was displayed using a 2D animation and a numerical counter that depicts the joint angle. Moreover, a *Color-Coded* mechanism helped them to identify if they are within or outside the reach of the *Target* that helped them to perform the range of motion exercise as accurately as possible.

Investigating different forms and modalities of *Feedback*, Cavalcanti et al. were able to identify multiple aspects which might help to design future rehabilitation applications [11]. For example, they were able to show that audio feedback was well perceived by users because it did not interfere with other visual information. However, the efficacy of audio feedback depends on the environment and may not work in clinical practice, e.g., due to disruption caused by external sound. *Text Feedback* was perceived better than *Image Feedback*. However, it was reported that it is critical that the display duration of the text is not too short to be able to understand the message clearly. For *Image-based Feedback*, the authors used a gif illustration that failed to convey the exact beginning and ending of a movement. *Scores*, on the other hand, helped to increase *Adherence*, i.e., to continue playing the game. Overall, Cavalcanti et al. reported that all forms of *Feedback* helped in some way to make correct movements and have the potential to motivate users in a physical rehabilitation setting.

Overall, the current state of research makes it difficult to draw generalizing conclusions. Many factors influence the choice and suitability of certain *Visual Guidance* elements. What worked well for one application might not work at all for another. Our main insights can be summarized as follows:

- Even for non-game-like applications, the use of AR/MR still provides a certain fascination and helps to keep users engaged. Still, if this is just a rather short-dated novelty effect or can be something that is sustained over time, remains to be seen.
- There is some evidence that there is a closer relation between specific types of *Visual Guidance* elements and certain *Medical Purposes*, such as *Gait & Balance* being addressed through *Obstacle* elements. However, again, the current body of work does not provide systematic studies of such aspects but base this conclusion on the overall evaluation of an application.
- There is growing evidence that simple video-based tele-rehabilitation approaches can not compete with AR/MR approaches, which should provide an interesting opportunity for future business models, as currently tele-rehabilitation and tele-fitness courses dominate the home market.

### 4.2. Implications for Output Technology

**Figure 9:**
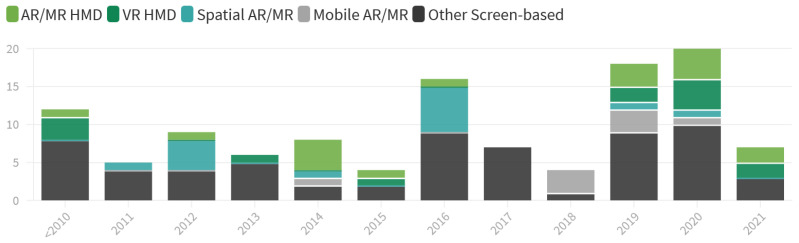
Used *Output Technologies* over the years.

The choice of *Output Technology* is seemingly unrelated to the publication date, as seen in Figure 9. A relatively low-tech *Screen* remains the predominant choice of display across the entire period under review. In total, *Screen-based* approaches account for 56% of output devices, while *Head-Mounted Displays* (HMD) are only used in 26% of the applications. The immense technological advances over the last 10 years for HMDs and in particular *AR/MR-HMDs*, including lighter hardware, more user-friendly interfaces, and increased immersive potential due to greatly improved specs, have only very recently seemed to emerge in the field of physical rehabilitation. From 2019 on, the number of such applications has risen and reached some levels of consistency in the amount of use - surprisingly not to the detriment of *Screen-based* approaches, which remained the most-used technology even in 2019 and 2020.

**Figure 10:**
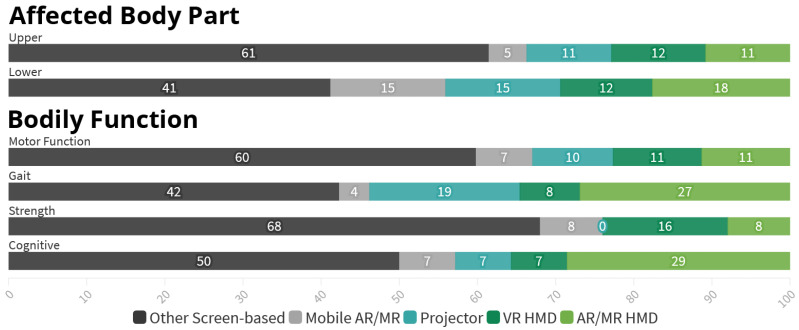
Differences in used *Output Technology* in relation to *Affected Body Part* and *Bodily Function*. Numbers represent percentages.

A certain level of influence on the *Output Technology* can be found regarding the *Affected Body Part* as well as *Bodily Function* as shown in Figure 10. However, upon closer inspection of the relevant data, we could not find a clear pattern that would explain, for example, the heavier reliance on *Screen-based* approaches for upper body parts compared to *Lower Body* parts. In addition, we also could not find any influence of the potential *Added Value* on the choice of *Output Technology*.

**Reasoning behind Technology Choices**: To gain a better understanding of the reasoning behind technology choices for *Output Technology*, we conducted an additional in-depth qualitative analysis among the most recent references from our data-set, published in 2019 and after. The analysis showed that only a fraction of the considered references provides any justification for the selection of the *Output Technology*. These justifications then differ again between the type of visualization technology chosen, AR/MR or VR, and the type of hardware selected, e.g., *HMD* or *Screen-based*.

*The choice of VR*: The use of VR, for example, is being discussed controversially. As a major disadvantage of VR, it is argued that patients do not receive feedback from the real world, which can lead to great insecurity and even health risks, especially for stroke patients who often suffer from balance problems [39,50,51]. In other papers, the separation of the patient from his real environment is considered to be a major advantage, because it avoids the patient being too distracted by the real environment [52,53] or allows the patient to be immersed in a completely virtual world, which can be particularly designed to support the exercises [43].

*The choice of AR/MR*: There are also differing opinions on whether and where AR/MR is suitable for use, some of which may be caused by unclear definitions of AR and/or MR. For example, Theunissen et al. claim that a property of MR is the filtering out of real visual information, which can then lead to safety risks due to the shielding form the real world, similar to VR [50]. However, most consider AR/MR to have the great advantage of allowing patients to be immersed in a virtual environment while still being able to perceive the real world [54]. It is noteworthy that no disadvantage of AR was explicitly mentioned in the selected papers.

*The choice of Screens*: The main reasons given for selecting either *Mobile Phones* or other *Screens* were that these are inexpensive and commercially available. In addition, patients are usually familiar with their operation [15,50]. Reference was also made to other work that has shown that tele-rehabilitation has proven itself effective in the past [42] and especially that *Mobile Phones* and other *Screens* are commonly found in households. One disadvantage of screens mentioned was the more difficult hand-eye coordination and split attention, since the visual attention cannot be on both, the own body and the screen simultaneously [34].

*The choice of HMDs*: *HMDs* primarily offer an advantage in representing the depth of motion in 3-dimensional space [38,48]. They are also significantly less expensive than having to set up a complete multisensory room [53]. A major disadvantage of *HMDs* is the impairment of head movement or an entire range of motion due to the inherent weight of the *HMD* and patients not being accustomed to carrying anything on their heads during movements or exercises [50].

A general implication to be taken from this analysis is that the choice of output technology depends on specific characteristics of the patient’s *Condition* and age, e.g., limitations in *Gait* functionality, *Balance*, and prior experience using technology [37,51]. Further, the scientific aim of the research plays an important role in the selection of technology. For example, some papers identify research gaps and target them specifically [43], which may have increased the focus on emerging AR/MR technology in recent years.

### 4.3. Research Opportunities

Based on our analysis, we identified a number of trends and open issues in AR/MR research within the field of *Physical Rehabilitation*, which we summarize below and which we think can help to guide future research:

- The analyzed literature reveals a strong focus on rehabilitation following *Neurological* conditions, especially *Stroke*. This may be due to research grants rather being provided to researchers who try to tackle such universal problems. Still, it would help if more research would then try to generalize the findings to other *Medical Conditions*. In particular, research dealing with AR/MR-based interventions following **Injury** or physical **Disabilities** is severely lacking.
- Research on *Upper Limb* is both more prevalent in general and more diverse, routinely focusing on individual joints and muscles, which at least can be partly explained through the importance of the *Hands* (and related body parts) for everyday activities and independence. Contrary, a majority of **Lower Limb** research targets *Gait or Balance*. The research focused on the use of AR/MR technology in rehabilitation and exercises of individual joints and muscles of the *Lower Limb* remains scarce. Additionally, research aimed at muscle **Strength** is severely underrepresented as well, in particular for *Lower Limb*.
- Regarding the utilized *Output Technology*, *Screens* still account for almost half of all display types used, even in most recent years. In contrast, there is ample research opportunity for more advanced AR/MR technologies such as **HMDs** and **Spatial-AR** through projection. *HMDs* might have an advantage for home-based exercise, but *Spatial-AR* could be installed in facilities and provide much of the same experience without the drawbacks of a (currently) heavy-weight head-worn device.
- The *Visual Guidance* elements **Virtual Coach** and on-body **Overlays** are relatively seldom types of demonstration, although the existing research shows promising results (e.g., [48,49]). While both *Virtual Coaches* and on-body *Overlays* are quite complex to realize and require a tracking of the environment or body, we think there is an opportunity here, in particular in combination with modern *AR/MR-HMDs* and their integrated inside-out tracking functionality.
- Furthermore, there is a lack of research explicitly comparing the effectiveness and advantages of selected **Visual Guidance elements**. While a few references did compare elements [14,28,30], they only do so aimed at very specific use cases. Therefore, this literature review cannot provide more guidance towards the usefulness and appropriateness of certain elements. Still, our review can serve as an inspirational starting point for practitioners and other researchers alike.

### 4.4. Limitations and Weaknesses

There are some limitations and potential weaknesses of this literature review that could be addressed in future research.

- Firstly, in this scoping literature review, information was gathered from different studies and sources without assessing or weighing the quality, accuracy, and validity of the papers. Instead, the focus was on collecting evidence to provide an overview of the research topic and assist researchers. Therefore, no assertions or suggestions can be made about the feasibility of the employed visualisations, nor is possible to derive a fair general comparison between them.
- Furthermore, the review was exploratory, employing an open-coding process. Therefore, the chosen categories, codes, and assignments could be considered arbitrary. In order to mitigate this factor, all steps leading to and including the final coding process were performed by at least two independent researchers and discussed with a third reviewer before a final call was made.
- The selection of search keywords is, as with every literature review, a topic for debate. We deliberately did not impose a predefined set of keywords on the search process, but rather applied an iterative process, adding relevant keywords along the way.
- Lastly, within this review, we identified distinct applications. Occasionally, this led to an inclusion of several such applications presented within a single reference or by the same research group. Although distinct, these routinely share many similarities, such as using the same technology, thereby distorting results. Therefore, the results do not accurately describe the amount and quality of research in each category. In order to limit this bias, we did not include papers describing an identical system, but only the one best representing it.

### 4.5. Strengths

Besides the above-mentioned limitations, our approach offers a number of strengths summarized in this section.

- The review address the broad topic of the use of Augmented and Mixed Reality applications in several areas of medical rehabilitation and for multiple conditions and affected body parts. Further, it takes into consideration a broad spectrum of sources, employing different methods and study designs. It is therefore able to provide an overview of the extent, range and nature of currently evolving areas of research as well as summarize research findings.
- By doing this, it also aids to identify trends, needs and research gaps to aid in future research.
- The review further provides a first taxonomy to classify the research area. The advantage of the exploratory approach employing an open-coding method lies in its bottom-up construction of categories and items based on actual research and relevant items within the sources.
- Additionally, several in-depth qualitative comparisons of selected recent studies were conducted, to provide a better understanding of the reasoning behind technology choices for Output Technology, this paper aims to further guide future research and experiment set-ups.

## 5. Conclusions

We analyzed 114 applications presented in 91 papers within the field of AR/MR research regarding physical exercises in medical rehabilitation. Employing an open coding process, we derived a taxonomy that distinguishes between different *Patient Types*, the *Medical Purpose* as well as the *Interaction Design* of proposed applications. In particular, the latter explores the relationship between *Output Technologies*, *Application Types*, *Input Technologies* and *Visual Guidance* elements. We identified 13 distinct, reoccurring abstract types of elements providing *Visual Guidance*, which we classified further into the three subgroups *Guided Interaction*, *Demonstrated Interaction* and *Feedback*. A deeper analysis of the selected data-set revealed the positive relationship between elements and the *Medical Purpose* as well as some insights into the effectiveness of certain *Visual Guidance* elements. We also presented qualitative evidence for researchers’ reasons for selecting certain *Output Technologies*. In summary, the taxonomy provides an overview of relevant attributes of AR/MR applications for physical rehabilitation. It bridges the gap between a medical/physiological perspective (*Patient Type*, *Medical Purpose*) and the Technology and *Interaction Design*. We are confident that it can serve as a reference for new research and practitioners alike, who aim to develop new approaches or get an understanding of existing work in a specific subarea. In particular, the set of *Visual Guidance* elements can serve as an inspiration for future *Interaction Designs*. The taxonomy also revealed the predominant motivation to develop technological solutions for physical rehabilitation process; the two biggest problems, based on what has been addressed in research, seem to be the lack of autonomy (or reliance on the presence of a therapists) as well as the lack of motivation (which often might be co-related with the absence of a therapist). Most researchers do not want to replace the therapist but either see the need for additional physical exercise or think that additional technology would help therapists to focus more on individual patients. The lack of motivation is mainly tackled by *Game-like Application* designs or gamification elements, such as *Performance Feedback*. Although our focus was on AR/MR applications, which are nowadays often naturally associated with *HMDs*, such as a Microsoft HoloLens device, most applications still use screens in a variety of form factors to provide visual guidance. Even though some researchers acknowledge the difficulties of split-attention and the less immersive experience of such *Screen-based* approaches, the wide availability, low cost, and technical limitations of *HMD*-based approaches (i.e., limited field of view) may help to explain this situation.

Given that, we pointed out a variety of research opportunities. In particular, we want to highlight the following three areas we see as critical for future research: (1) applying and studying the efficacy of advanced AR/MR technologies to overcome the limitations of *Screen-based* approaches; (2) atudies comparing and understanding the efficacy of different forms of *Visual Guidance* and *Application Types*; (3) the tackling of currently underrepresented *Body Parts* or *Bodily Functions*.

Finally, we want to stress the importance of multidisciplinary research in this area. Only a combination of experts from the medical field as well as HCI (design, technology, user research) is able to address the taxonomy as a whole and make sensible technology and medical application choices.

## Data Availability

The datasets used in this study are available on request from the corresponding author.

## Abbreviations

The following abbreviations are used in this manuscript:

ACM	Association for Computing Machinery
AR	Augmented Reality
HCI	Human Computer Interaction
HMD	Head-Mounted Display
IEEE	Institute of Electrical and Electronics Engineers
MR	Mixed Reality
VR	Virtual Reality
WHO	World Health Organization

## Appendix A

**Table A1:** Overview of the selected references—author names A–H, part 1/2, Abbr. in Table A7. Distinct applications within a reference are marked with lowercase letters.

1st Author	Ref.	Med. Condition	Aff. Body Part	Bodily Function	Added Value
Achanccaray	[52]	NE, NS	UL, FH	MF	AC, AH, EF
Agopyan	[55]	NE, NS	LL, KN	MF, GB	AC, EF
Alamri	[32] a	NE, NS	UL, FH	MF	AH, AU, PA
Alamri	[32] b	NE, NS	UL, FH	MF	AH, AU, PA, PF
Alexandre	[56]	NE, NS, CO	UL, FH	MF, ST	AC, AH, AU, PA, PF
Aung	[57] a	NE, NS	UL	MF, ST	AH, AU, EF, PF
Aung	[57] b	NE, NS	UL, FH, SH, EL	MF, ST	AH, AU, EF, PF
Aung	[58]	NE, NS	UL, FH, SH, EL	MF	AU, EF
Bakker	[23]	NE, NS	BO	CG	AH, AU, EF, PF
Baran	[59]	NE, NS	UL, FH	MF	AH, AU, PA, PF
Bell	[42]	IS	LL, KN	MF	AC, AH, AU, PF
Bouteraa	[27]	NE, NS	UL, FH	MF	AH, PA, PF
Broeren	[60]	NE, NS	UL	MF	AH, AU, PF
Broeren	[61]	NE, NS	UL, FH	MF, CG	AH, AU, EF
Burke	[62] a	NE, NS	UL	MF, ST	AH, AU, EF, PF
Burke	[62] b	NE, NS	UL	MF, ST, CG	AH, AU, PF
Camporesi	[63]	CO	UL	MF	AH, AU, PA, PF
Cavalcanti	[11]	NE	UL, SH, EL	MF	AC, AH, AU, EF, PA, PF
Chang	[51]	NE, NS	LL, KN	MF, GB	AH, EF, PA, PF
Chen	[43]	NE, NS	LL	GB	AU
Colomer	[14] a	NE, NS	UL, FH	MF	AH, AU, EF, PF
Colomer	[14] b	NE, NS	UL, FH, SH, EL	MF	AH, AU, EF, PF
Corrêa	[64]	CO	UL, FH, EL	MF	AH, EF
Da Gama	[65]	NE, NS	UL, FH, SH, EL	MF	AC, AH, AU, EF, PF
Dancu	[66]	NE, NS	UL, FH	MF	AC, AU, PF
David	[67]	NE, NS	UL, SH, EL	MF	AH, AU, EF, PF
Desai	[68] a	NE, NS	LL	GB	AH, AU, EF, PA, PF
Desai	[68] b	NE, NS	UL, EL	MF	AH, AU, EF, PA, PF
Desai	[68] c	NE, NS	UL, EL	MF, CG	AH, AU, EF, PA, PF
Dinevan	[33] a	NE, NS	UL, FH	MF	AH, AU, PF
Dinevan	[33] b	NE, NS	UL, SH	MF, ST	AH, AU, PF
Enam	[69]	NE, NS	LL, AN	GB	AH, EF
Fonteyn	[47]	NE	LL	MF, GB	AU, PA, PF
Garcia	[13]	IS	LL, AN	MF	AH, AU, EF, PF
Gauthier	[70]	NE, NS	UL, FH, SH, EL	MF, ST, CG	AH, AU, EF
Grimm	[71]	NE, NS	UL, FH, SH, EL	MF	AC, AH, PA
Guo	[72]	NE, NS	UL, LL	MF	AH, AU, EF, PA, PF
Hacioglu	[73]	CO	UL, FH	MF	AH
Halic	[74]	CO	UL, FH	MF, ST	AH, AU, PA, PF
Han	[49]	CO	UL	MF	AC, AU, PF
Mohd Hashim	[53]	NE, NS	LL	MF, CG	AH, AU
Held	[36]	NE, NS	LL	MF, GB, CG	AU, PF
Hoermann	[75]	NE, NS	UL, FH	MF, CG	AH, AU, EF
Hoermann	[76]	NE, NS	UL, FH	MF	AH, EF

**Table A2:** Overview of the selected references—author names A–H, part 2/2, Abbr. in Table A7. Distinct applications within a reference are marked with lowercase letters.

1st Author	Ref.	Output Tech.	Application Type	Visual Guidance	Input Tech.
Achanccaray	[52]	HVR	TB, MI	OB, RE	AD
Agopyan	[55]	SC	TB	SE	MBT, AD
Alamri	[32] a	HVR	TB	TA, OB	MBT
Alamri	[32] b	HVR	TB	OB, PT	MBT
Alexandre	[56]	SC	GA	TA, OB, RC, GS	SBT, HD
Aung	[57] a	SC	GA	OB, RC, SE, GS	MBT, AD
Aung	[57] b	SC	GA	TA, OB, DI, SE, GS	MBT, AD
Aung	[58]	SC	TB, MI	TA, PT, OV	MBT, AD
Baker	[23]	HAR	GA	TA, OB, RC, RE, GS	SBT
Baran	[59]	SC	TB	OB, PT	MBT, AD
Bell	[42]	SC	TB	RE, GS, CC	MBT, SBT
Bouteraa	[27]	SC	GA	TA, OB, SE, GS	OT, AD
Broeren	[60]	SC	GA	TA, OB, GS	HD
Broeren	[61]	SC	GA	TA, OB, RC, OS, GS	HD
Burke	[62] a	SC	GA	TA, OB, RC, GS	MBT
Burke	[62] b	SC	TB	TA, OB, GS, CC	MBT
Camporesi	[63]	SC	TB	VT, RE, SE, GS	OT, MBT
Cavalcanti	[11]	SC	GA	TA, OB, RC, RE, GS, TE	OT
Chang	[51]	HAR	GA	TA, OB, PT, GS, TE	SBT, AD
Chen	[43]	SC	GA	OB, DI, OV, SE, GS, TE	OT
Colomer	[14] a	SAR	GA	TA, OB, GS, TE	OT
Colomer	[14] b	SAR	GA	OB, RC, GS	OT
Corrêa	[64]	SC	TB	OB, CC	MBT
Da Gama	[65]	SC	GA	TA, OB, RE, GS, TE	OT
Dancu	[66]	SC	TB	TA, VT, RE, SE, GS	OT
David	[67]	SC	GA	TA, GS	OT
Desai	[68] a	SC	GA	OB, SE, GS	OT
Desai	[68] b	SC	GA	OB, SE, GS	OT
Desai	[68] c	SC	GA	TA, OB, SE, GS, TE	OT
Dinevan	[33] a	SC	GA	OB, RC, GS	MBT
Dinevan	[33] b	SC	GA	OB, RC, GS, TE	MBT, AD
Enam	[69]	SAR	TB	TA	AD
Fonteyn	[47]	SAR	TB	TA, OS	MBT, AD
Garcia	[13]	MAR	GA	TA, OB, RC, PT	MBT
Gauthier	[70]	SC	GA	TA, OB, OS, SE, GS	OT
Grimm	[71]	SC	TB	TA, OB	SBT, HD
Guo	[72]	MAR	GA	TA, OB, PT, GS, TE	OT
Hacioglu	[73]	SC	GA	OB, PT	OT
Halic	[74]	SC	GA	OB, OS, GS	SBT
Han	[49]	HAR	TB	RE, OV	OT, SBT, AD
Mohd Hashim	[53]	HVR	GA	OB, GS, TE	OT
Held	[36]	HAR	GA	TA, OB, PT, OS, DI, TE, CC	SBT
Hoermann	[75]	SC	GA, MI	OB, GS, CC	OT, AD
Hoermann	[76]	SC	MI	RE	AD

**Table A3:** Overview of the selected references—author names I–Sr, part 1/2, Abbr. in Table A7. Distinct applications within a reference are marked with lowercase letters.

1st Author	Ref.	Med. Condition	Aff. Body Part	Bodily Function	Added Value
Ines	[77]	NE, NS	UL, FH, EL	MF	AH, AU, EF, PA
Jin	[78]	NE, NS	LL	GB, ST	AC, AH, AU, PA
Jung	[79]	NE, NS	LL, AN	MF, GB, ST	
Keskin	[44] a	NE, NS	UL, FH	MF	AH, AU, EF, PA
Keskin	[44] b	NE, NS	UL, FH, EL	MF	AH, AU, EF, PA, PF
Khan	[39]	CO	UL, LL	GB	AC, AU
King	[80]	NE, NS	UL, FH	MF	AH, AU, EF
Klein	[81]	NE, NS	UL, SH	MF	EF
Kloster	[21]	IS	BO		AH, AU, PF
Koroleva	[82] a	NE, NS	LL	MF, GB	AC, PA, PF
Koroleva	[82] b	NE, NS	UL, FH	MF, ST	AC, PA, PF
Kowatsch	[24]	CO	UL, LL, BO	ST	AH, AU, EF, PF
LaPiana	[83]	NE, NS	UL, FH	MF, ST	AH, AU, EF
Lee	[84]	NE, NP	LL	GB	AH, AU, EF
Liu	[85] a	NE, NS	UL, FH	MF, ST	AH, AU, PF
Liu	[85] b	NE, NS	UL, FH	MF, ST	AH, AU, PF
Liu	[85] c	NE, NS	UL, FH, SH, EL	MF, ST	AC, AH, AU, PF
Liu	[26] a	NE, NS	LL	GB	AU, PF
Liu	[26] b	NE, NS	UL, FH	MF	AH, AU
Lledó	[86]	NE, NS	UL	MF	AC, AH, AU, PA, PF
Loureiro	[87]	NE, NS	UL, FH, SH, EL	MF	AC, AH
Luo	[88]	NE, NS	UL, FH	MF	AH
Manuli	[89]	NE, NS	LL	MF, GB, ST, CG	AH, EF, PF
Monge	[90]	NE, NS, IS, CO	LL	MF	AH, AU, EF, PF
Mostajeran	[48]	CO		GB	AU, PA, PF
Mostajeran	[37]	CO		MF, GB, CG	AC, AH, AU, PA, PF
Mouraux	[91]	NE, IS	UL	MF	AH, AU
Muñoz	[92]	NE, IS	UL, FH, SH, EL	MF	AC, AH, AU, PA, PF
Nanapragasam	[93]	NE, NS	LL	GB	
Pachoulakis	[40]	NE, NP	UL, FH, SH, EL, LL	MF	AC, AH, AU, EF, PA, PF
Paredes	[54]	CO	LL	GB, CG	AH, AU, EF, PA, PF
Park	[31]	NE, NS	UL, FH	MF	AH, AU
Phongamwong	[46]	NE, NS	LL	MF, GB	AC, PA, PF
Ramirez	[94]	NE, NS	LL	ST	AH, AU, EF
Regenbrecht	[95]	NE, NS	UL, FH	MF	AH
Saraee	[96]	NE, NS, IS, CO	UL	MF, ST	AH, EF, PA
Seyedebrahimi	[34]	NE, NS, NP	UL, FH	MF, CG	EF
Shen	[41]	NE, NS	UL, FH	MF	AC, AH, AU, PF
Sodhi	[30] a	CO	UL, FH	MF	AC, AU
Sodhi	[30] b	CO	UL, FH	MF	AC, AU
Sodhi	[30] c	CO	UL, FH	MF	AC, AU
Sodhi	[30] d	CO	UL, FH	MF	AC, AU, PF
Song	[97] a	NE, NS	UL	MF	AH, AU, EF, PF
Song	[97] b	NE, NS	UL	MF, CG	AH, AU, PF
Sousa	[12]	IS	UL, SH, EL	MF	AC, AH, AU, EF, PA, PF
Sror	[98]	NE, NS	UL, EL	MF	AC, EF, PF

**Table A4:** Overview of the selected references—author names I–Sr, part 2/2, Abbr. in Table A7. Distinct applications within a reference are marked with lowercase letters.

1st Author	Ref.	Output Tech.	Application Type	Visual Guidance	Input Tech.
Ines	[77]	SAR	GA	TA, OB	MBT
Jin	[78]	HAR	GA	TA, OB, PT, OS, DI, GS	OT, AD
Jung	[79]	HVR	TB	RE, SE	AD
Keskin	[44] a	SC	TB	TA, OB	OT
Keskin	[44] b	SC	GA	OB, RC, GS	OT
Khan	[39]	HVR	TB	RE	MBT
King	[80]	SC	GA	TA, OB, RC, GS	MBT
Klein	[81]	SC	TB, MI	OV	MBT, AD
Kloster	[21]	HVR	GA	TA, TE, CC	SBT
Koroleva	[82] a	SC	TB	PT, OS, GS	OT
Koroleva	[82] b	SC	GA	OB, PT, GS, CC	OT
Kotwasch	[24]	HAR, SC	TB	VT, TE	SBT
LaPiana	[83]	HVR	GA	TA, OB, GS	MBT
Lee	[84]	HAR	TB	RE, TE	
Liu	[85] a	SC	TB	TA, OB, RC, GS, CC	MBT
Liu	[85] b	SC	GA	OB, RC, GS	MBT
Liu	[85] c	SC	TB	TA, OB, RC, PT, GS, CC	MBT
Liu	[26] a	SC	TB	SE	MBT, AD
Liu	[85] b	SC	MI	RE, OV	MBT, AD
Lledó	[86]	SC	TB	TA, OB, GS, CC	HD
Loureiro	[87]	SC	TB	TA, OB, PT	HD
Luo	[88]	HAR	TB	OB	SBT
Manuli	[89]	SC	TB	TA, OS	AD
Monge	[90]	MAR	GA	VT, GS	SBT, AD
Mostajeran	[48]	HAR	TB	VT	OT
Mostajeran	[37]	HAR	GA	TA, OB, PT, VT	OT, AD
Mouraux	[91]	SC	GA, MI	TA, OB, SE, CC	OT
Muñoz	[92]	SC	GA	OB, DI, RE, SE, GS	OT
Nanapragasam	[93]	SC		TA, SE	MBT, AD
Pachoulakis	[40]	SC	TB	PT, DI, RE, OV, GS, TE	OT
Paredes	[54]	HAR	GA	TA, OB, OS, GS, TE	OT, SBT, AD
Park	[31]	HVR	GA	TA, OB, GS	OT
Phongamwong	[46]	SC	TB	SE, GS	MBT, AD
Ramirez	[94]	MAR	GA	TA, OB, GS, TE	SBT
Regenbrecht	[95]	SC	GA	OB	OT
Saraee	[96]	SC	TB	TA, OB, RC, PT	HD
Seyedebrahimi	[34]	SAR, SC	GA	TA, RC, GS	MBT
Shen	[41]	HAR	GA	TA, OB, DI, OV, GS, TE, CC	MBT, SBT
Sodhi	[30] a	SAR	TB	DI, OV	OT
Sodhi	[30] b	SAR	TB	DI, OV	OT
Sodhi	[30] c	SAR	TB	TA, PT, OV, CC	OT
Sodhi	[30] d	SAR	TB	DI, OV, CC	OT
Song	[97] a	MAR	GA	TA, OB, RC, GS	OT
Song	[97] b	MAR	GA	TA, OB, GS, TE	OT
Sousa	[12]	SAR	TB	TA, RC, PT, OV, GS, CC	MBT
Sror	[98]	SC	TB	TA, TE	OT, HD

**Table A5:** Overview of the selected references—author names Sy–Z, part 1/2, Abbr. in Table A7. Distinct applications within a reference are marked with lowercase letters.

1st Author	Ref.	Med. Condition	Aff. Body Part	Bodily Function	Added Value
Syed Ali Fathima	[99]	CO	UL, FH, SH, EL	MF	AH, AU, PA, PF
Tang	[28]	IS	UL, SH	MF, ST	AC, AU, PF
Theunissen	[50] a	NE, NS	LL	MF, GB	AC, AH, AU, EF, PA, PF
Theunissen	[50] b	NE, NS	LL	MF, GB	AC, AH, AU, EF, PA, PF
Theunissen	[50] c	NE, NS	LL	MF, GB	AC, AH, AU, EF, PA, PF
Thikey	[100]	NE, NS	LL, AN, KN	MF, GB	AC, AH, PA, PF
Timmermans	[35] a	NE, NS	LL	GB	AH, AU, PF
Timmermans	[35] b	NE, NS	LL	GB	AH, AU, PF
Timmermans	[35] c	NE, NS	LL	GB	AH, AU, PF
Toh	[29]	NE, NS	UL, FH	MF	AH, AU, EF, PF
Trojan	[101] a	NE, NS, CO	UL, FH	MF	AH, AU, PA, PF
Trojan	[101] b	NE, NS, CO	UL, FH	MF	AH, AU, PA, PF
Trojan	[101] c	NE, NS, CO	UL, FH	MF	AH, AU, PA, PF
Trojan	[101] d	NE, NS, CO	UL, FH	MF	AH, AU, PA, PF
Velloso	[22]	CO		ST	AC, AU, EF, PA, PF
Vidrios-Serrano	[45]	NE, NS	UL	MF	AH, AU, PA
Voinea	[102]	NE, NS	UL, FH	MF	AH, AU
Wei	[103]	NE, NS	UL	MF	AH, AU, PA, PF
Xue	[104]	NE, NS, IS, CO	UL, FH, SH, EL	MF, ST	AC, AH, AU, PA, PF
Yang	[105]	CO	UL, LL	MF	AC, AU, PA, PF
Yeh	[106]	NE, NS	UL, FH, SH	MF	AC, AH, AU, PA
Yeo	[38]	CO	UL, SH	MF	AC, AH, AU, PA, PF
Yu	[15] a	CO	UL	MF, ST	AC, AU, PF
Yu	[15] b	CO	UL	MF, ST	AC, AU, PF

**Table A6:** Overview of the selected references—author names Sy–Z, part 2/2, Abbr. in Table A7. Distinct applications within a reference are marked with lowercase letters.

1st Author	Ref.	Output Tech.	Application Type	Visual Guidance	Input Tech.
Syed Ali Fathima	[99]	SC	GA	TA, OB, GS	OT
Tang	[28]	SC	TB	TA, PT, DI, OV, SE, GS, TE, CC	MBT
Theunissen	[50] a	SC	GA	GS	MBT, SBT, AD
Theunissen	[50] b	MAR	GA	TA, OB, GS	SBT
Theunissen	[50] c	SC	TB		SBT
Thikey	[100]	SC	TB	TA, SE, GS, CC	MBT
Timmermans	[35] a	SAR	TB	OS	AD
Timmermans	[35] b	SAR	TB	TA	AD
Timmermans	[35] c	SAR	GA	OB	AD
Toh	[29]	SC	GA	TA, OB, RC, OS, DI, GS, TE	MBT
Trojan	[101] a	HAR	MI	TA, RE, CC	OT
Trojan	[101] b	HAR	MI	TA, RE	OT
Trojan	[101] c	HAR	GA, MI	TA, OB, RE	OT
Trojan	[101] d	HAR	MI	OB, RE	OT
Velloso	[22]	SC	TB	DI, RE, OV, SE, GS, TE, CC	OT, SBT
Vidrios-Serrano	[45]	HAR	TB	TA, OB, RC, CC	MBT, HD
Voinea	[102]	HVR	TB, MI	VT, RE, SE	
Wei	[103]	SC	TB	SE, GS, CC	AD
Xue	[104]	MAR	GA	TA, OB	OT
Yang	[105]	HVR	TB	TA, OB, OV, SE	SBT
Yeh	[106]	SC	TB	TA, OB	MBT
Yeo	[38]	SC	TB	TA, DI, RE, SE, GS, TE	OT
Yu	[15] a	HVR	TB	TA, OB, PT, DI, RE, SE, CC	OT
Yu	[15] b	HVR	TB	PT, SE, CC	OT, MBT

**Table A7:** Abbreviations for Table A1, Table A2, Table A3, Table A4, Table A5, Table A6.

Category	Abbreviation		
Medical Condition	NE = Neurological	NS = Stroke	NP = Parkinson
	IS = Injury/Surgery	CO = Other Condition	
Affected Body part	UL = Upper Limb	FH = Hand/Fingers/Wrists	SH = Shoulder
	EL = Elbow	LL = Lower Limb	AN = Ankle
	KN = Knee	BO = Other	
Bodily Function	MF = Motor function	GB = Gait/Balance	ST = Strength
	CG = Cognitive function		
Added Value	AH = Adherence	PA = Post-session Analysis	AC = Accuracy
	AU = Autonomy	PF = Performance Feedback	EF = Effort
Output Technology	HVR = VR HMD	HAR = AR/MR HMD	SAR = Spatial AR/MR
	MAR = Mobile AR/MR	SC = Other Screen-based	
Application Type	GA = Game-based	TB = Task-based	MI = Mirror Therapy
Visual Guidance	TA = Target	OB = Object	RC = Racket/Cursor
	PT = Path	OS = Obstacle	DI = Direction
	VT = Virtual Coach	RE = Recording	OV = Overlay
	SE = Self-Evaluation	GS = Score/Graph	TE = Text
	CC = Color-code		
Input Technology	OT = Optical Tracking	MBT = Marker-based Tracking	AD = Additional Device
	HD = Haptic Device	SBT = Sensor-based Tracking	
